# Supplementary material for: Loss of Kat2a enhances transcriptional noise and depletes acute myeloid leukemia stem-like cells
Source: eLife. 2020 Jan 27;9:e51754. doi: 10.7554/eLife.51754 (PMC7039681; doi:10.7554/eLife.51754)
Supplement: Transparent reporting form [file elife-51754-transrepform.docx]

***eLife’s* transparent reporting form**

We encourage authors to provide detailed information *within their submission* to facilitate the interpretation and replication of experiments. Authors can upload supporting documentation to indicate the use of appropriate reporting guidelines for health-related research (see EQUATOR Network), life science research (see the [BioSharing Information Resource](https://biosharing.org/)), or the [ARRIVE guidelines](http://www.plosbiology.org/article/info:doi/10.1371/journal.pbio.1000412) for reporting work involving animal research. Where applicable, authors should refer to any relevant reporting standards documents in this form.

If you have any questions, please consult our Journal Policies and/or contact us: [editorial@elifesciences.org](mailto:editorial@elifesciences.org).

**Sample-size estimation**

- You should state whether an appropriate sample size was computed when the study was being designed
- You should state the statistical method of sample size computation and any required assumptions
- If no explicit power analysis was used, you should describe how you decided what sample (replicate) size (number) to use

Please outline where this information can be found within the submission (e.g., sections or figure legends), or explain why this information doesn’t apply to your submission:

Justification of animal usage detailed in the Materials and Methods (page 19). We did not perform an explicit power calculation to determine the numbers of animals used in primary AML transplant. We were instead guided by prior knowledge of the MLL-AF9 leukaemia model and estimated that <6 animals would reveal a significant prolongation of mean survival of 10 days given the low SD of the control group.

- You should report how often each experiment was performed
- You should include a definition of biological versus technical replication
- The data obtained should be provided and sufficient information should be provided to indicate the number of independent biological and/or technical replicates
- If you encountered any outliers, you should describe how these were handled
- Criteria for exclusion/inclusion of data should be clearly stated
- High-throughput sequence data should be uploaded before submission, with a private link for reviewers provided (these are available from both GEO and ArrayExpress)

Please outline where this information can be found within the submission (e.g., sections or figure legends), or explain why this information doesn’t apply to your submission:

Uploading of High-throughput data on GEO : “Data deposition” in the Materials and Methods section (page 30). Reviewer’s token has been generated for the same and will be shared at the time of submission. Relevant information is present in each figure legend.

**Statistical reporting**

- Statistical analysis methods should be described and justified
- Raw data should be presented in figures whenever informative to do so (typically when N per group is less than 10)
- For each experiment, you should identify the statistical tests used, exact values of N, definitions of center, methods of multiple test correction, and dispersion and precision measures (e.g., mean, median, SD, SEM, confidence intervals; and, for the major substantive results, a measure of effect size (e.g., Pearson's r, Cohen's d)
- Report exact p-values wherever possible alongside the summary statistics and 95% confidence intervals. These should be reported for all key questions and not only when the p-value is less than 0.05.

Please outline where this information can be found within the submission (e.g., sections or figure legends), or explain why this information doesn’t apply to your submission:

“Statistical analysis” in Materials and Methods section (page 27). Details regarding statistical test, exact values of N, tests used and p values alongside precision measures are indicated in figure legends, and further described in the statistical analysis in methods section.

**Group allocation**

- Indicate how samples were allocated into experimental groups (in the case of clinical studies, please specify allocation to treatment method); if randomization was used, please also state if restricted randomization was applied
- Indicate if masking was used during group allocation, data collection and/or data analysis

Please outline where this information can be found within the submission (e.g., sections or figure legends), or explain why this information doesn’t apply to your submission:

Blinding procedures for analysis of animal experiments detailed in the Materials and Methods section (page 19).

- We encourage you to upload relevant additional data files, such as numerical data that are represented as a graph in a figure, or as a summary table
- Where provided, these should be in the most useful format, and they can be uploaded as “Source data” files linked to a main figure or table
- Include model definition files including the full list of parameters used
- Include code used for data analysis (e.g., R, MatLab)
- Avoid stating that data files are “available upon request”

Please indicate the figures or tables for which source data files have been provided:

The relevant R scripts and corresponding data files have been provided for Figures 3C, 3D, 4A, Figure 3– figure supplement 1B, Fig.4 – figure supplement 2E and 2F. Processed D3E output files used for Prism GraphPad analysis in Figures 3A, 3B, 3E, 3F and 5C, and in Figure 5D are also provided. Individual experimental values are displayed in most panels; exceptions are stacked column graphs in Figure 1-figure supplement 1F, Figure1-figure supplement 2E, and Figure 6E, for which source data files are given.
